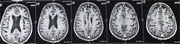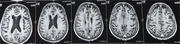# Prostatic malignancy with paraneoplastic subacute encephalitis with unique imaging characteristics

**DOI:** 10.1002/alz.086745

**Published:** 2025-01-09

**Authors:** Mridula Singh, Suman Kumar Sahu, Suman Kushwaha, Siddharth Maheshwari, Ashwin Kumar Panda, Alisha Mustafa

**Affiliations:** ^1^ IHBAS, New Delhi, New Delhi India; ^2^ IHBAS, New Delhi, New Delhi, Delhi India

## Abstract

**Background:**

Prostatic malignancy with paraneoplastic subacute encephalitis –A rare syndrome

**Method:**

We present a case of 76 year old male without any previous comorbidity and addiction who manifested a rapid neuropsychiatric decline with a frontotemporal syndrome over a period of 6 months. He was anemic and cerebrospinal fluid study showed 10 cells with lymphocytic predominance. The extensive workup of csf for infection, malignancy revealed nothing. His workup for metabolic, neuroinfection and stroke came out negative. His PSA came 4 times the upper limit and MRI brain showed T2 hyperintensity in left temporal lobe with punctate contrast enhancement and atrophy of right temporal lobe with enlarged temporal horn. His MRI pelvis showed PIRADS 4 lesion highly suggestive of malignancy. His paraneoplastic markers came negative. As our institute being neuropsychiatric one, we had to refer the patient to multispecialty hospital. The patient succumbed to illness within two months after referral from our institute while undergoing treatment for the malignancy.

**Result:**

Prostatic malignancy causing paraneoplastic rapidly progressive dementia with unusual MRI features.

**Conclusion:**

Limbic encephalitis presents with short term memory loss, complex partial seizures and neuropsychiatric complaints. Our case presented subacutely as frontotemporal syndrome. The signal abnormalities in the mesial temporal lobes without contrast enhancement are the typical MRI findings. In our case patient had extensive involvement of temporal lobe with contrast enhancement, so likey an overlap between limbic encephalitis and encephalomyelitis. To the best of my knowledge none of the case in past had this MRI feature.